# Health Benefits of Air Purification: A Systematic Review

**DOI:** 10.1021/envhealth.5c00836

**Published:** 2026-06-22

**Authors:** Xiaowei Xue, Yixuan Jiang, Weijie Qian, Renjie Chen, Haidong Kan

**Affiliations:** † School of Public Health, Shanghai Institute of Infectious Disease and Biosecurity, Key Lab of Public Health Safety of the Ministry of Education, NHC Key Lab of Health Technology Assessment, 12478 Fudan University, Shanghai 200032, China; ‡ 614406 Amway (China) Co., Limited, Guangzhou, Guangdong 510000, China; § National Center for Children’s Health, Children’s Hospital of Fudan University, Shanghai 201102, China

**Keywords:** air purification, indoor air quality, cardiorespiratory health, neurocognitive function, cost-effectiveness

## Abstract

Air pollution is a critical global public health challenge that poses a wide range of health threats. As people spend most of their time indoors, household air pollution is a significant yet often overlooked source of exposure. Existing evidence suggests that air purification is a promising intervention to mitigate these risks. This systematic review synthesized evidence from studies published between January 1990 and August 2025 on the effectiveness of air purifiers for improving the indoor air quality and mitigating health impacts. We also assess the cost-effectiveness and identify major limitations in current research. The findings demonstrate that air purifiers significantly reduce indoor pollution levels, leading to substantial respiratory and cardiovascular benefits, particularly in high-risk populations. However, evidence of neurocognitive outcomes remains limited. Despite heterogeneity in study designs, air purifiers represent a potentially cost-effective strategy for mitigating the adverse effects from air pollution. Standardized device certification, supportive policies to improve access, and continued research to optimize intervention strategies are warranted for the future.

## Graphical abstract

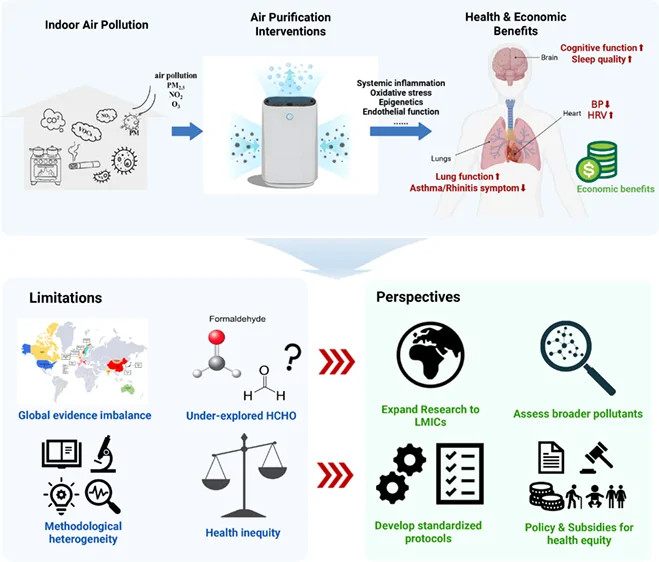

## 1 Introduction

Globally, air pollution remains a critical public health challenge.^1,2^ According to the most recent Global Burden of Disease (GBD) 2023 estimates, air pollution remains a leading environmental risk factor for premature death and disability-adjusted life years (DALYs) globally.^3^ Fine particulate matter (PM_2.5_) continues to rank among the top risk factors for total DALYs worldwide, reportedly accounting for 8.2% (95% uncertainty interval: 6.7%, 9.7%) of DALYs, underscoring its substantial contribution to the global burden of diseases.^3^ While policy often focuses on outdoor ambient pollution, indoor air quality poses a threat that is at least as significant.^4^ Individuals spend approximately 90% of their time indoors,^5–7^ where indoor air pollution levels can surpass outdoor concentrations, particularly in poorly ventilated environments. This is due to the infiltration of ambient pollutants and emissions from indoor sources such as cooking, smoking, volatile organic compounds from various products.^8,9^ This “indoor penalty” is amplified in vulnerable populations, including children,^10^ pregnant individuals,^10^ and those with pre-existing conditions such as asthma or chronic obstructive pulmonary disease (COPD).^11^ Therefore, it is essential to address the health impacts of indoor air pollution by controlling indoor air quality to reduce the associated disease burden.

While various methods and approaches are available to control indoor pollutant levels, strategies such as improving ventilation systems and controlling pollution sources are often constrained by limited resources. Air purification systems, particularly portable air cleaners equipped with high-efficiency particulate air (HEPA) filters, have emerged as a viable solution. These devices are estimated to reduce indoor PM_2.5_ by 50–90% through mechanical filtration, electrostatic precipitation, or hybrid technologies and may show promising potential as an intervention.^12^ A growing body of research has explored the health benefits of air purifiers in mitigating air pollution but significant heterogeneity in study design, intervention duration, and technology type has made it difficult to draw consistent conclusions.

Therefore, this systematic review comprehensively assessed the effectiveness of air purifier interventions in improving indoor air quality and their associated health benefits. By synthesizing evidence from studies with different intervention durations, air purifier types, and health outcomes, this review aimed to provide recommendations regarding the most effective air purifiers for different settings as well as the optimal duration of intervention. The findings provide guidance for the large-scale implementation of air purification strategies and the reduction of air pollution-related health inequities.

## 2 Methodology

### 2.1 Search Strategy and Protocol

A systematic literature search was conducted in PubMed and Web of Science for peer-reviewed, English-language articles published between January 1, 1990, and August 31, 2025. The search utilized a combination of keywords and Medical Subject Headings (MeSH) terms related to “air purifier,” “air purification,” “air cleaner,” “air filter,” “high-efficiency particulate air filter,” “air pollution,” “health,” “cardiovascular,” “respiratory,” “cardiopulmonary,” “cardiorespiratory,” “neurocognitive,” “neurodevelopment,” “cognitive function,” “mortality,” “morbidity,” “health benefit,” “randomized intervention study,” “randomized crossover trial,” and “randomized controlled trial.” Boolean operators (AND/OR) and truncation symbols (*) were applied to capture variations in terminology.

The systematic review was conducted following the Preferred Reporting Items for Systematic Reviews and Meta Analyses (PRISMA) guidelines for reporting.^13,14^

### 2.2 Study Inclusion/Exclusion Criteria

The following inclusion and exclusion criteria were applied: (1) We included randomized intervention studies, as well as cost-effective or health benefit studies related to air purification, and excluded chamber-based experimental studies where air quality was not managed by a portable device. All types of randomized intervention studies were included, such as randomized crossover trials, randomized clinical trials, randomized parallel-group studies, and randomized self-controlled studies, without restriction on blind methods and intervention durations. To ensure the integrity of blinding, a “sham” condition was typically utilized to provide a controlled comparison without reducing indoor pollutant concentrations. The configuration of a sham unit depends on the underlying technology. In mechanical filtration systems, such as HEPA purifiers, researchers typically remove the internal filter. Conversely, in trials using active purification technologies such as ionizers, electrostatic precipitators (ESPs), or photocatalytic oxidation (PCO) devices, the active electrical or ionizing components are deactivated. Regardless of the technology, the device fan continues to operate at identical speeds to ensure that participants remain blinded to the intervention. (2) The study population comprised individuals across all age groups exposed to air pollution. We included both healthy populations and vulnerable subgroups, such as children, elderly individuals, and pregnant women. Additionally, specific occupational groups (e.g., office workers and taxi drivers) were also incorporated into the analysis. (3) We included interventions involving various indoor air purification technologies (HEPA, ionizers, and photocatalysis). Studies focusing on personal protective equipment (e.g., face masks) or source control (e.g., changing cookstoves) were excluded. (4) The health end points assessed in this review included clinical indicators, biomarkers, symptom scores, and disease status related to cardiovascular, respiratory, cardiopulmonary, and neurocognitive systems, as well as indicators of cost-benefit or disease burden.

### 2.3 Eligible Study Selection and Data Extraction

After removing duplicates, two authors independently screened all remaining studies’ titles, abstracts, and the full texts according to the inclusion and exclusion criteria. Then, they extracted the information on eligible studies independently. Discrepancies were resolved through discussion.

Using a predefined standardized form, the following key information on each study was extracted: authors, year of publication, study region, study period, study design, study population, sample size, type of air purifier, setting, intervention and washout period, pollutant type, outcomes, and study findings.

## 3 Results and Discussion

### 3.1 Summary of Included Literature

A total of 3979 records were identified through searching the database. After removing 237 duplicates, 3742 records remained for screening, and 3016 were excluded based on title and abstract screening. Of the remaining 726 records, 613 were further excluded after an in-depth review of the full text. Finally, 113 articles were included in this review (Figure 1).

**Figure 1:**
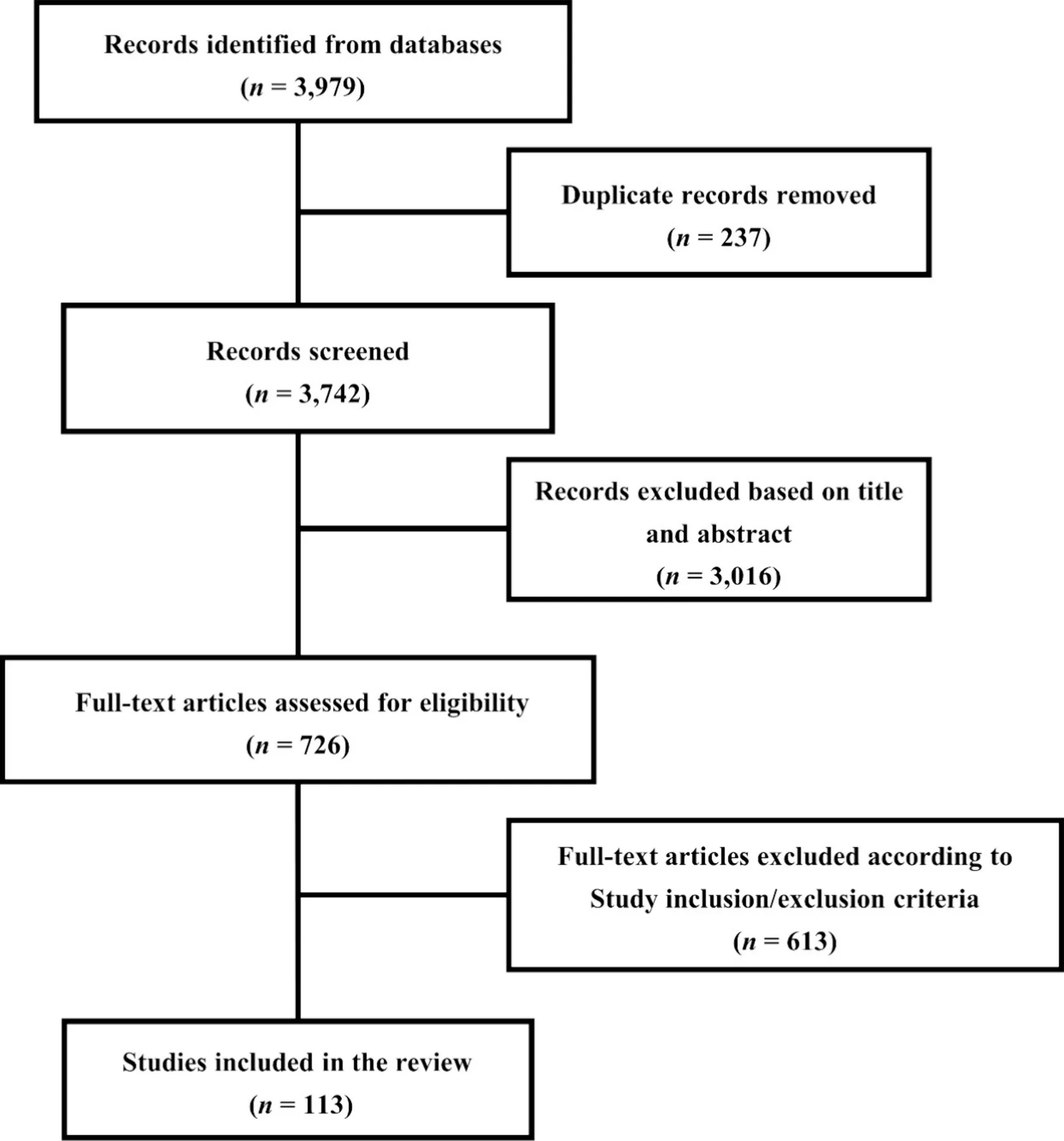
Flowchart for study selection.

Specifically, the identified studies were mainly conducted in East Asia, North America, and Northern Europe (Figure 2). The study population encompassed pregnant women, children, older adults, and adults, spanning a wide age range (1–103 years). Sample sizes varied from 9 to 2123 participants (median: 47), with one study including 136,000 children across 290 schools. The majority of studies incorporated into this review (*n* = 106) were randomized intervention studies. Seven studies calculated the cost-effectiveness or health benefits attributable to air purification by constructing mathematical models rather than conducting field research. The majority of studies (*n* = 96, 85%) utilized HEPA filters. Other air cleaners included ESPs (*n* = 5), ionization purifiers (*n* = 3), nonthermal plasma (NTP) purifiers (*n* = 1), negative ion generators (*n* = 1), PCO purifiers (*n* = 1), photoelectrochemical oxidation (PECO) purifiers (*n* = 1), and in-vehicle CO_2_ filtration systems (*n* = 1), with an additional 4 studies employing unspecified air purifier types. Generally, HEPA and ESP were the predominant technologies used for particulate matter reduction, while active technologies such as PCO and ionizers were primarily targeted toward gaseous pollutants or bioaerosols. Detailed technical specifications for each study are provided in Tables 1–4. Interventions varied in duration, encompassing both short- and medium-to-long-term periods. The duration of interventions across all included studies ranged from 1.5 h to 18 months. Included studies focused on the health benefits associated with reducing levels of indoor particulate matter (primarily PM_2.5_), gaseous pollutants (nitrogen dioxide, NO_2_; carbon dioxide, CO_2_; volatile organic compound, VOC), black carbon, dust, various airborne allergens (e.g., dust mites, fungi, pollen, cat allergens), benzene compounds, secondhand smoke, pathogen-laden aerosols, and phthalates.

**Figure 2:**
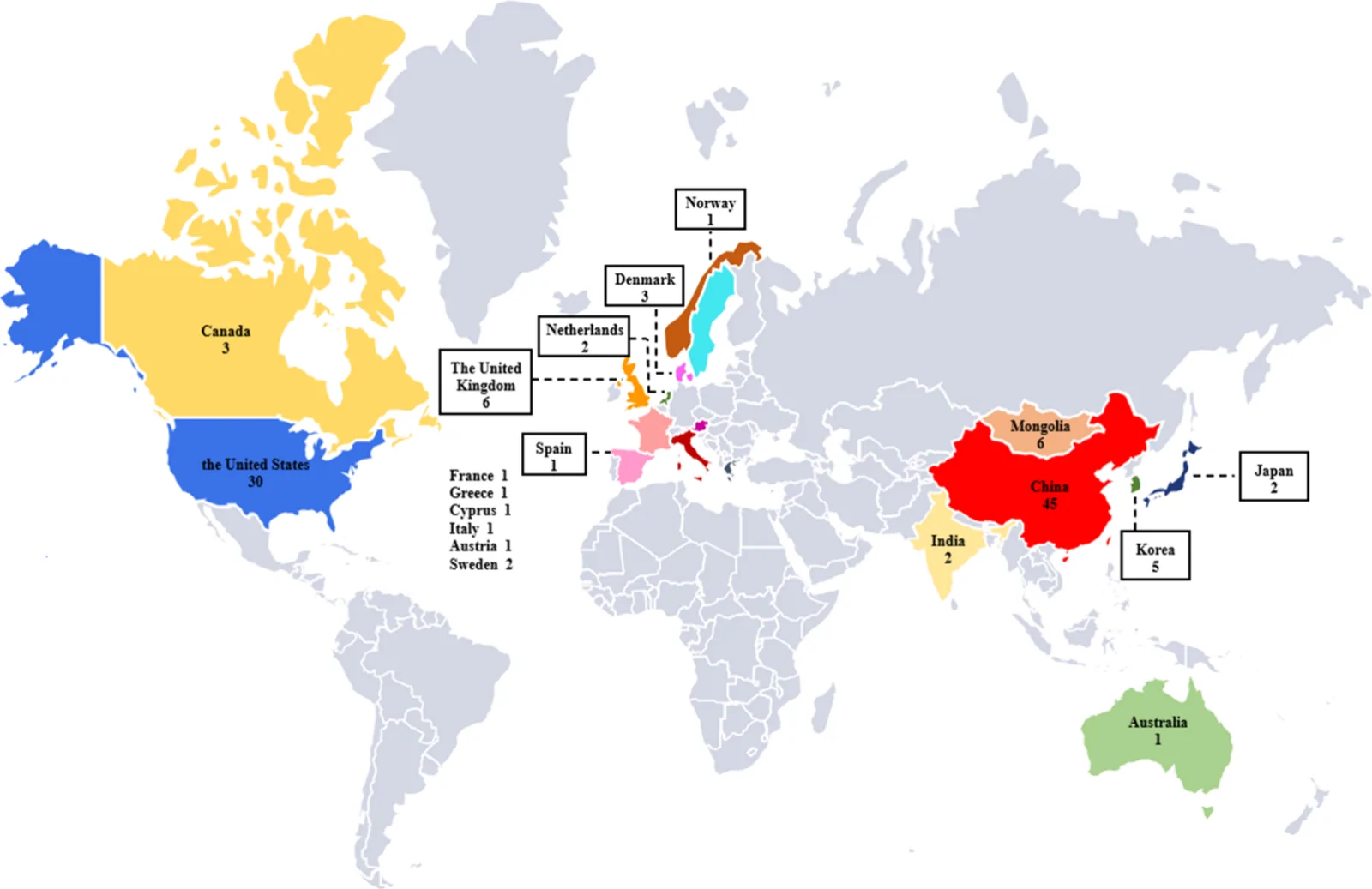
Global geographical distribution of included studies on effects of indoor air purifiers use on health.

**Table 1:** Characteristics of Included Studies on Respiratory Systems^a^

**author(s)**	**study region**	**study period**	**study design**	**study population**	**sample size**	**purifier type**	**setting**	**intervention duration**	**pollutant(s)**	**outcome(s)**	**result(s)**
Yoda et al.^62^	Hanshin, Japan	2018.11–2019.2	randomized double-blind crossover trial	healthy adults	32	HEPA	living room	4 weeks per phase, 4 week washout	endotoxin in indoor PM_2.5_ and PM_2.5–10_	FVC, FEV_1_, FEV_1_/FVC, MMEF, PEF, FVC(V_50_/V_25_), FeNO	no significant change
Yoda et al.^63^	Osaka and Hanshin, Japan	2018.11–2019.2	randomized double-blind crossover trial	healthy adults	32	HEPA	living room	4 weeks per phase, 4 week washout	PM_2.5_	FVC, FEV_1_, FEV_1_/FVC MMEF, PEF, FVC(V_50_/V_25_), FeNO	no significant change
Li et al.^15^	Yulin, China	2016.6–2018.9	randomized parallel-group trial	patients with allergic rhinitis sensitive to artemisia pollen	90	HEPA	bedroom	4 week intervention	pollen	VAS score, nasal symptoms, allergic symptom scores, RQLQ scores	significant improvement
Zhao et al.^64^	Beijing, China	2021.11–2022.4	randomized crossover trial	healthy adults	68	HEPA	dormitory	4 days per phase, 16 day washout	airborne microorganisms	FVC, FEV_1_ FEF_25_, FEF_50_, FEF_75_, FEF_25–75_, PEF	significant improvement
Gherasim et al.^16^	Strasbourg, France	2017.12.6–2018.2.19	randomized crossover trial	patients with feline asthma	24	HEPA	standardized environment	intervention duration not specified, 3 week washout	cat allergen	early and late asthmatic responses, bronchial reactivity, FEV_1_	significant improvement
Skulberg et al.^47^	Oslo, Norway	January to March	randomized crossover trial	workers with respiratory symptoms	80	electrostatic air purifier	office	3 week intervention	dust	nasal dimensions, PEF	significant improvement
Lee et al.^23^	Incheon, South Korea	2018.9.3–2018.10.27	randomized double-blind crossover trial	elementary school students with asthma	30	HEPA	living room	3 weeks per phase, 2 week washout	PM_2.5_	asthma severity, lung function, PEFR, FeNO, airway inflammation, urinary microbiome and phthalate levels	significant improvement
Lei et al.^65^	Mengzhou, China	2021.4–2021.12	randomized crossover trial	healthy elementary school students	79	HEPA	classroom, children’s bedroom	76 days per phase, 88 day washout	PM_2.5_	FEV_1_, PEF, FEV_1_/FVC, FVC, FEF_25–75_, MEF_75_, MEF_50_, MEF_25_, FeCO, FeNO, COHb, EBC metabolome	significant improvement
Warner et al.^52^	London, UK		randomized double-blind crossover trial	children with allergic asthma	20	ionizing air purifier	living room, bedroom	6 weeks per phase, no washout period	dust mite allergen	PEFR, symptom scores	no significant change
James et al.^33^	Cincinnati, US	2015.10–2017.8	randomized double-blind crossover trial	children with asthma	43	HEPA	residence	1 month per phase, 1 month washout	PM_2.5_, BC, UV-absorbing particulate matter, fungal spores	asthma control, quality of life scores	significant improvement
van der Heide et al.^25^	Groningen, Netherlands	1997.9–1998.4	randomized double-blind crossover trial	children with asthma sensitive to cat or dog allergens	20	HEPA	living room, bedroom	3 months per phase, no washout period	animal allergens in dust	lung function, airway hyperresponsiveness, peak flow variability	significant improvement
Yang et al.^66^	Beijing, China	2019.3.18–2019.4.26	randomized double-blind crossover trial	healthy children	125	HEPA	classroom, dormitory	3 days per phase, 1 month washout	PM_10_, PM_2.5_, PM_1_	lung function, biomarkers of inflammation and oxidative stress	significant improvement
Gent et al.^24^	Connecticut and Massachusetts, US	2015.9–2019.4	randomized double-blind crossover trial	children with persistent asthma	126	HEPA	residence	4 weeks per phase, 1 week washout	NO_2_, PM_2.5_	days with asthma symptoms	no significant change
Cui et al.^26^	Shanghai, China	2017.2.14–2017.4.24	randomized double-blind crossover trial	children with mild-to-moderate asthma	43	HEPA	bedroom	2 weeks per phase, 2 week washout	PM_2.5_	FeNO, spirometry, impulse oscillometry, PEF	significant improvement
Kim et al.^34^	Incheon, South Korea	2018.9–2018.10	randomized double-blind crossover trial	children with asthma	26	HEPA	bedroom or main living space	3 weeks per phase, 1 week washout	PM_2.5_	PEFR, FeNO	significant improvement
Xu et al.^35^	New York, US	2007.10–2008.4	randomized double-blind crossover trial	children with asthma	30	HEPA	bedroom	6 weeks per phase, 6 week washout	PM_10_, VOC, CO, CO_2_	EBC, PEF	significant improvement
Khadar et al.^18^	New South Wales, Australia	2023.4.7–2023.10.26	randomized crossover trial	residents of an aged care facility	135	HEPA	rooms in a residential aged care facility	3 months per phase, 1 week washout	pathogen-containing aerosols	incidence of ARIs, time to first infection, emergency department visits, hospitalizations, medical consultations for ARI	significant improvement
Reisman et al.^32^	New York and Maryland, US	November to March	randomized double-blind crossover trial	patients with a multiyear history of rhinitis and/or asthma during fall-winter	32	HEPA	bedroom	4 months per phase, no washout period	particulate matter ≥ 0.3 μm	symptom/medication scores	significant improvement
Antonicelli et al.^51^	Ancona, Italy	1988.10–1989.2	randomized double-blind crossover trial	patients with dust mite allergy	9	HEPA	residence	2 months per phase, no washout period	dust mites	symptom scores, bronchial hyperreactivity	no significant change
van der Heide et al.^17^	Groningen, Netherlands		randomized parallel-group trial	patients with allergic asthma	45	HEPA	living room, bedroom	6 month intervention	airborne allergenic particles	lung function, airway hyperresponsiveness, skin tests	significant improvement
Francis et al.^44^	Manchester, UK		randomized parallel-group trial	adult asthmatic patients allergic to cats and/or dogs	30	HEPA	living room, Bedroom	12 month intervention	dust	combined asthma outcome, lung function	significant improvement
Wood et al.^49^	Maryland, US		randomized parallel-group trial	patients with cat allergy	35	HEPA	bedroom	3 month intervention	airborne allergens, dust	nasal symptom score, chest symptom score, sleep disturbance, peak flow rates, rescue medication use	no significant change
Sulser et al.^27^	Germany	1999.8–2000.10	randomized parallel-group trial	children with asthma and cat/dog allergy with significant exposure	30	HEPA	living room, bedroom	1 year intervention	levels of pet allergens in filter and bulk dust samples	lung function	no significant change
Walker et al.^67^	Alaska, Montana and Navajo Nation; US	two winters	randomized parallel-group trial	children ≤ 5 years old in homes with wood stoves	416	HEPA	room with wood stove	two winter seasons	PM_2.5_	incidence of lower respiratory tract infections	no significant change
Lanphear et al.^43^	Cincinnati, US		randomized parallel-group trial	children with asthma exposed to secondhand smoke	215	HEPA	main activity space, bedroom	1 year intervention	secondhand smoke	asthma exacerbation, asthma symptoms, FeNO	significant improvement
Jhun et al.^48^	Northeast (3 cities), US	2013–2014	cluster-randomized parallel-group trial	children with asthma	25	HEPA	classroom	1 year intervention	PM_2.5_, BC	lung function, asthma symptoms	improved peak flows no change in FEV1 and asthma symptom
Phipatanakul et al.^29^	Northeast (41 urban schools), US	2015–2020	cluster-randomized parallel-group trial	students with active asthma	236	HEPA	classroom	10 month intervention	PM_2.5_, PM_2.5–10_, BC, allergens in air and settled dust	asthma symptom days, school absence days, composite asthma severity index, healthcare utilization, lung function	no significant change
Butz et al.^36^	Baltimore, US		randomized parallel-group trial	children with asthma living with smokers	126	HEPA	living room, bedroom	6 month intervention	PM_2.5_, PM_2.5–10_, airborne nicotine, urinary cotinine	asthma symptom-free days	significant improvement
Luo et al.^46^	Guangzhou, China	2016.8–2017.2	randomized parallel-group trial	children with mild allergic asthma	38	HEPA	bedroom	6 month intervention	PM, dust, mite allergens in dust	asthma control status, FeNO	significant improvement
Park et al.^45^	Seoul and Gwangju, South Korea	October to November	randomized double-blind crossover trial	adults with allergic rhinitis	44	HEPA	living room, bedroom	6 week intervention	PM_2.5_, PM_10_	improvement in allergic rhinitis symptoms, medication scores, quality of life, VAS scores	significant improvement
Kadalayil et al.^31^	Isle of Wight, UK	2019.9–2021.7	randomized parallel-group trial	adults with mild-to-moderate persistent, uncontrolled asthma	50	HEPA	living room, bedroom	18 month intervention	PM_2.5_	asthma and rhinitis symptom, sleep quality of life	significant improvement
Woo et al.^20^	Baltimore, US	2014.4–2019.1	randomized parallel-group trial	former-smoking adult COPD patients	94	HEPA	living room, bedroom	6 month intervention	PM_2.5_, NO_2_	SGRQ score	significant improvement
Hansel et al.^21^	Baltimore, US	2014.4–2019.1	randomized parallel-group trial	former-smoking adult COPD patients	94	HEPA	living room, bedroom	6 month intervention	PM_2.5_, NO_2_	changes in SGRQ over six months	significant improvement
Fawzy et al.^19^	Baltimore, US	2014.4–2019.1	randomized parallel-group trial	former-smoking adult COPD patients	116	HEPA	living room, bedroom	6 month intervention	PM_2.5_, PM_10_, UFP	IL-8, IL-6, IL-1β, TNF-α, IFN-γ, VCAM-1, ICAM-1, P-selectin, sCD40L, 8-OHdG, 8-iso-PGF_2α_	no significant change
Kouis et al.^37^	Cyprus and Greece	2021.2–2021.6 2021.9–2021.12	randomized crossover trial	children with asthma	182	HEPA	classroom, home	3 months per phase	PM_10_	asthma control status	significant improvement
Drieling et al.^38^	Yakima Valley, US	2015.7–2019.2	randomized parallel-group trial	rural latino children with asthma	75	HEPA	living room, bedroom	1 year intervention	PM_2.5_	ACT score, asthma symptoms, unscheduled clinical care use	significant improvement
Park et al.^39^	Fresno, US	2015.4–2015.7	randomized parallel-group trial	children with asthma and/or allergic rhinitis	17	HEPA	living room, bedroom	12 week intervention	PM_2.5_	asthma control level, peak expiratory flow, nasal symptom scores	significant improvement
Morgan et al.^40^	Boston, Chicago, Dallas, New York City, Seattle, Tacoma, Tucson; US	1998.8–1999.7	randomized parallel-group trial	children with atopic asthma	937	HEPA	bedroom	1 year intervention	dust, various airborne allergens, secondhand smoke, mold	days of most severe symptoms in the 2 weeks preceding the interview, spirometry, PEF, medication and healthcare use	significant improvement
Kaviany et al.^41^	Baltimore, US	2018.9–2020.1	randomized parallel-group trial	children with asthma	127	HEPA	main activity space, bedroom	12 week intervention	PM_2.5_	asthma severity, asthma control level	significant improvement
Fong et al.^50^	Isle of Wight, UK	2019.9–2021.7	randomized parallel-group trial	adults with mild-to-moderate persistent, uncontrolled asthma	50	HEPA	living room, bedroom	18 month intervention	PM_2.5_, PM_10_, benzene, NO_2_	change in ACQ-6 and AQLQ scores, lung function, bronchial hyperreactivity, FeNO, PEF	no significant change
Huang et al.^42^	Florida, US	1993.7–1994.6	randomized parallel-group trial	children with perennial rhinitis due to mold allergy	75	air purifier	bedroom	2 year intervention	mold	symptom scores	significant improvement
Luo et al.^22^	Guangzhou, China	2015.10–2016.2	randomized parallel-group trial	patients with allergic rhinitis	32	HEPA	bedroom	4 month intervention	allergens in dust, PM_1_, PM_2.5_, PM_10_	allergic rhinitis quality of life scores	significant improvement
Rao et al.^30^	Austin, US	2015.3–2017.4	randomized double-blind crossover trial	adults with varying degrees of nasal or ocular allergy symptoms, some with asthma	46	PECO	bedroom	4 week intervention	airborne organic compounds and microorganisms	total nasal and ocular allergy symptom score	significant improvement
Moreno-Rangel et al.^28^	McAllen, US	2019.6–2019.11	randomized crossover trial	children with asthma	13	HEPA	bedroom	7 day intervention	PM_2.5_	overall health, asthma control level	significant improvement
Weisboeck-Erdheim et al.^68^	Salzburg and Upper Austria, Austria	2022.11–2023.3	cluster-randomized parallel-group trial	office workers	150	NTP	office	5 month intervention	airborne microorganisms	upper respiratory tract infections incidence and severity	significant improvement

a Abbreviations: HEPA, high-efficiency particulate air; PECO, photoelectrochemical oxidation; NTP, nonthermal plasma; PM_2.5_, particulate matter with an aerodynamic diameter ≤2.5 μm; PM_10_, particulate matter with an aerodynamic diameter ≤ 10 μm; PM_1_, particulate matter with an aerodynamic diameter ≤ 1 μm; PM_2.5–10_, particulate matter with an aerodynamic diameter between 2.5 and 10 μm; BC, black carbon; UV, ultraviolet; VOC, volatile organic compound; CO, carbon monoxide; CO_2_, carbon dioxide; NO_2_, nitrogen dioxide; UFP, ultrafine particles; FVC, forced vital capacity; FEV_1_, forced expiratory volume in 1 s; FEV_1_/FVC, the ratio of FEV_1_ to FVC; MMEF, maximal midexpiratory flow; PEF/PEFR, peak expiratory flow (rate); V_50_, flow rates at 50% of FVC; V_25_, flow rates at 25% of FVC; FEF25, forced expiratory flow at 25% of FVC; FEF_50_, forced expiratory flow at 50% of FVC; FEF_75_, forced expiratory flow at 75% of FVC; FEF_25–75_, forced expiratory flow at 25–75% of FVC; MEF_25_, maximal expiratory flow at 25% of vital capacity; MEF_50_, maximal expiratory flow at 50% of vital capacity; MEF_75_, maximal expiratory flow at 75% of vital capacity; FeNO, fraction of exhaled nitric oxide; FeCO, fraction of exhaled carbon monoxide, COHb, carboxyhemoglobin; EBC, exhaled breath condensate; VAS score, visual analogue scale score; RQLQ, rhinoconjunctivitis quality of life questionnaire; ARI, acute respiratory infection; SGRQ, St. George’s respiratory questionnaire; ACT score, asthma control test score; ACQ-6, asthma control questionnaire-6; AQLQ, asthma quality of life questionnaire; IL-8, interleukin-8; IL-6, interleukin-6; IL-1β, interleukin-1β; TNF-α, tumor necrosis factor-α; IFN-γ, interferon-γ; VCAM-1, vascular cell adhesion molecule-1; ICAM-1, intercellular adhesion molecule-1; sCD40L, soluble CD40 ligand; 8-OHdG, 8-hydroxy-2′-deoxyguanosine; 8-iso-PGF_2α_, 8-iso-prostaglandin F_2α_.

**Table 2:** Characteristics of Included Studies on Cardiopulmonary Systems^a^

**author(s)**	**study region**	**study period**	**study design**	**study population**	**sample size**	**purifier type**	**setting**	**intervention duration**	**pollutant(s)**	**outcome(s)**	**result(s)**
Sun et al.^78^	Beijing, China	2017.12–2018.4	randomized double-blind crossover trial	healthy adults	29	HEPA	dormitory	pollution waves, >2 week washout	PM_2.5_	BP, FeNO, lung function, inflammatory cytokines, DNA methylation	significant improvement
Wang et al.^53^	Beijing, China	2017.11–2018.4	randomized double-blind crossover trial	healthy adults	54	HEPA	dormitory	1 week per phase, 2 week washout	PM_2.5_	BP, lung function, FeNO, circulating biomarkers of platelet activation and blood coagulation, systemic oxidative stress	significant improvement
Zhao et al.^54^	Beijing, China	2017.11–2018.4	randomized double-blind crossover trial	healthy adults	29	HEPA	dormitory	pollution waves, >2 week washout	PM_2.5_	BP, lung function, FeNO, circulating biomarkers of platelet activation and blood coagulation, systemic oxidative stress	significant improvement
Wang et al.^56^	Beijing, China	2017.11–2018.4	randomized double-blind crossover trial	healthy adults	57	HEPA	dormitory	1 week per phase, >2 week washout	PAEs	BP, lung function, FeNO, circulating biomarkers of platelet activation and blood coagulation, systemic oxidative stress	significant improvement
Chen et al.^57^	Shanghai, China	2014	randomized double-blind crossover trial	healthy adults	35	HEPA	dormitory	48 h per phase, 2 week washout	PM_2.5_	circulating biomarkers of inflammation, coagulation, and vasoconstriction, lung function, BP, FeNO	significant improvement
Liu et al.^58^	Beijing, China	2018.6–2018.11	randomized double-blind crossover trial	healthy adults	56	negative ion air purifier	dormitory	1 week per phase, 2 week washout	PM_2.5_	FeNO, FVC, FEV_1_, BP, augmentation index, PWV, neutrophils, malondialdehyde, 8-isoprostane	no significant change
Weichenthal et al.^59^	Manitoba, Canada	2011	randomized double-blind crossover trial	residents of a first nations community	37	electrostatic air purifier	residence	1 week per phase, 1 week washout	PM_2.5_, BaP, BTEX, NO_2_	lung function, BP, endothelial function	significant improvement
Cui et al.^60^	Shanghai, China	2015.11.7–2015.12.13	randomized double-blind crossover trial	healthy nonsmoking adults	70	HEPA	dormitory	13 h per phase, 2 week washout	PM_2.5_	lung function, FeCO, BP, augmentation index, subendocardial viability ratio, HR, PWV, inflammatory and coagulation factors, oxidative stress markers	significant improvement
Dong et al.^84^	Beijing, China	2017.12–2018.3	cluster-randomized double-blind crossover trial	healthy children	44	ionizing air purifier	classroom	5 days per phase, 2 month washout	PM, BC	BP, HRV, HR, ST-segment elevation, FeNO, PEF, FEV_1_, MDA	improved lung function reduced HRV
Liu et al.^85^	Beijing, China	2017.12–2018.3	cluster-randomized double-blind crossover trial	healthy children	44	ionizing air purifier	classroom	5 days per phase, 2 month washout	PM, negative air ions	HRV, HR, lung capacity, FeNO, urinary metabolome	significant improvement
Guo et al.^86^	Chongqing, China	2020.1.6–2020.1.22	randomized double-blind crossover trial	healthy elderly	24	HEPA	room in aged-care center	2 days per phase, 12 day washout	PM_1_, PM_2.5_, PM_10_	BP, HR, FeNO, lung function, biomarkers of inflammation, coagulation, and oxidative stress	significant improvement
Shao et al.^88^	Beijing, China	2013.12–2014.3	randomized double-blind crossover trial	nonsmoking elderly with/without COPD	35	HEPA	living room, bedroom	2 weeks per phase, no washout	PM_2.5_	HRV, BP, lung function, systemic inflammation and oxidative stress biomarkers	no significant change
Karottki et al.^98^	Copenhagen, Denmark	2010.11–2011.5	randomized double-blind crossover trial	nonsmoking adults	48	HEPA	living room, bedroom	14 days per phase, no washout	PM_2.5_	BP, microvascular function, lung function, CRP, total cholesterol, HDL, LDL, triglycerides	no significant change
Zhang et al.^93^	Kaohsiung, China	2020–2023	randomized double-blind crossover trial	patients with asthma	65	PCO, filter air cleaner	living room	2 weeks per phase, 2 week washout	PM_1_, PM_2.5_, PM_4_, PM_10_, TSP, UFP, TVOC, NO_2_, SO_2_, CO, CO_2_	lung function, FeNO, respiratory symptoms, BP	no significant change
Day et al.^200^	Changsha, China	2014.12.1–2015.1.31	randomized double-blind parallel-group trial	office workers	86	HEPA, ESP	office, dormitory	5 week intervention	PM_2.5_, O_3_	biomarkers of oxidative stress, systemic inflammation, lung function, BP, PWV, SEVR, vWF	HEPA: no significant change ESP-HEPA: adverse effect

a Abbreviations: HEPA, high-efficiency particulate air; PCO, photocatalytic oxidation; ESP, electrostatic precipitator; PM_2.5_, particulate matter with an aerodynamic diameter ≤ 2.5 μm; PM_10_, particulate matter with an aerodynamic diameter ≤ 10 μm; PM_1_, particulate matter with an aerodynamic diameter ≤ 1 μm; PM_4_, particulate matter with an aerodynamic diameter ≤ 4 μm; PM, particulate matter; PAEs, phthalic acid esters; BaP, benzo­[a]­pyrene; BTEX, benzene, toluene, ethylbenzene, and xylenes; NO_2_, nitrogen dioxide; SO_2_, sulfur dioxide; CO, carbon monoxide; CO_2_, carbon dioxide; O_3_, ozone; BC, black carbon; TSP, total suspended particulates; UFP, ultrafine particles; TVOC, total volatile organic compounds; FVC, forced vital capacity; FEV_1_, forced expiratory volume in 1 s; PEF, peak expiratory flow; FeNO, fraction of exhaled nitric oxide; FeCO, fraction of exhaled carbon monoxide; BP, blood pressure, HR, heart rate, HRV, heart rate variability; PWV, pulse wave velocity, SEVR, subendocardial viability ratio; vWF, von Willebrand factor; MDA, malondialdehyde; CRP, C-reactive protein; HDL, high-density lipoprotein; LDL, low-density lipoprotein; COPD, chronic obstructive pulmonary disease.

**Table 3:** Characteristics of Included Studies on Cardiovascular Systems^a^

**author(s)**	**study region**	**study period**	**study design**	**study population**	**sample size**	**purifier type**	**setting**	**intervention duration**	**pollutant(s)**	**outcome(s)**	**result(s)**
Lyu et al.^72^	Beijing, China	2020.10–2020.11	randomized double-blind crossover trial	healthy adults	20	HEPA	print shop	7 days per phase, 14 day washout	printing shop particles	HRV	significant improvement
Xia et al.^69^	Xi’an, China	2020.6–2020.8	randomized double-blind crossover trial	healthy adults	38	HEPA	dormitory	36 h per phase, 7 day washout	PM_1_, PM_2.5_, PM_10_	BP, blood oxygen saturation, HRV	significant improvement
Chen et al.^80^	Shanghai, China	2014	randomized double-blind crossover trial	healthy adults	35	HEPA	dormitory	48 h per phase, 2 week washout	PM_2.5_	DNA methylation, circulating biomarkers	significant improvement
Li et al.^76^	Shanghai, China	2015.11–2015.12	randomized double-blind crossover trial	healthy adults	55	HEPA	dormitory	9 days per phase, 12 day washout	PM_2.5_	cortisol, cortisone, epinephrine, norepinephrine	significant improvement
Chen et al.^77^	Shanghai, China	2015.11–2015.12	randomized double-blind crossover trial	healthy adults	55	HEPA	dormitory	9 days per phase, 12 day washout	PM_2.5_	markers of inflammation, coagulation, and vasoconstriction	significant improvement
Li et al.^79^	Shanghai, China	2015.11–2015.12	randomized double-blind crossover trial	healthy adults	36	HEPA	dormitory	9 days per phase, 12 day washout	PM_2.5_	DNA methylation	significant improvement
Wen et al.^300^	Beijing, China	2017.11–2018.4	randomized double-blind crossover trial	healthy adults	54	HEPA	dormitory	1 week per phase, 2 week washout	PM_2.5_	inflammatory markers	significant improvement
Allen et al.^75^	British Columbia, Canada	2008.11–2009.4	randomized double-blind crossover trial	healthy adults	45	HEPA	main living room, bedroom	7 days per phase, no washout	PM_2.5_	microvascular endothelial function, CRP, IL-6, MDA, 8-isoprostane	improved endothelial function reduced inflammatory biomarkers
Kajbafzadeh et al.^74^	Vancouver, Canada	2011.12–2012.8	randomized double-blind crossover trial	healthy adults	68	HEPA	living room, bedroom	7 days per phase, no washout	PM_2.5_	CRP, IL-6, circulating endothelial cells	reduced CRP
Chen et al.^70^	Northern Taiwan, China	2017–2019	randomized crossover trial	healthy adults	86	CO_2_ filtration system	in-vehicle	3 modes (control, closed, open), each separated by one month	CO_2_	HR, BP, drowsiness	significant improvement
Chuang et al.^73^	Taipei, China	2013.1.1–2014.12.31	randomized double-blind crossover trial	healthy adults	200	HEPA	living room, bedroom	1 year per phase, no washout	PM_2.5_, VOC	BP, CRP, 8-OHdG, fibrinogen	significant improvement
Padró-Martinez et al.^201^	Somerville, US	2011.2–2012.11	randomized double-blind crossover trial	adults with a history of disease	20	HEPA	living room	21 days per phase, no washout	UFP	CRP, IL-6, TNF-RII, fibrinogen	reduced IL-6
Li et al.^83^	Mengzhou, China	2021.4–2021.12	randomized crossover trial	healthy children	79	HEPA	classroom, bedroom	76 days per phase, 88 day washout	PM_2.5_	BP, HR	significant improvement
Guo et al.^87^	Chongqing, China	2020	randomized double-blind crossover trial	elderly people	24	HEPA	room in aged-care center	48 h per phase, 12 day washout	PM_1_, PM_2.5_, PM_10_	BP, HR	improved HR
Liu et al.^89^	Beijing, China	2013.12–2014.3	randomized double-blind crossover trial	elderly people	35	HEPA	living room, bedroom	2 weeks per phase, no washout	PM_2.5_, BC	12h HRV, ABPM	significant improvement
Bräuner et al.^97^	Copenhagen, Denmark	2006.4.3–2006.4.7	randomized double-blind crossover trial	healthy nonsmoking elderly	41	HEPA	living room, bedroom	48 h per phase, no washout	UFP, PM_2.5_, PM_2.5–10_	MVF, BP, hemoglobin, erythrocytes, platelets, coagulation factors, P-selectin, serum amyloid A, CRP, fibrinogen, IL-6, TNF-α, malondialdehyde, urinary 8-iso-PGF_2α_	improved MVF
Morishita et al.^94^	Detroit, US	2014.10.21–2016.11.4	randomized double-blind crossover trial	nonsmoking adults	40	HEPA	bedroom, main living room	3 days per phase, 1 week washout	PM_2.5_	BP, aortic hemodynamics, PWV, HRV	improved BP
Chen et al.^90^	Beijing, China	2013.12–2014.3	randomized double-blind crossover trial	nonsmoking elderly	35	HEPA	living room, bedroom	2 weeks per phase, no washout	PM_2.5_, BC	biomarkers of myocardial injury	significant improvement
Liu et al.^92^	Beijing, China	2018.12.16–2019.6.21	randomized double-blind crossover trial	patients with stable CAD	24	HEPA	main family activity space	3 days per phase, > 14 day washout	PM_2.5_	inflammation and coagulation markers, plaque stability, lipids	significant improvement
Eom et al.^99^	Cheongju and Daejeon, Korea	2020.11–2021.2	randomized double-blind crossover trial	patients with CAD	38	HEPA	living room	2 weeks per phase, 2 week washout	PM_2.5_	BP, HRV, baroreflex sensitivity, autonomic function tests, endothelial function	significant improvement
Zhou et al.^71^	Beijing, China	2022	randomized double-blind crossover trial	office workers	40	HEPA	office	1 day (5 h) per phase, no washout	PM_2.5_	HRV, cognitive tests, electrodermal activity	significant improvement
Xia et al.^91^	Hong Kong, China	2017.11–2020.12	randomized parallel-group trial	elderly people	47	HEPA	main activity room	1 year intervention	PM_2.5_	BP, FMD, carotid intima-media thickness	significant improvement
Lin et al.^202^	Taipei, China	2009.3–2009.10	randomized crossover trial	healthy adults	60	HEPA	residence	two intervention periods (25 days each period)	PM_2.5_, VOCs	BP, HR	significant improvement
Raju et al.^95^	Baltimore, US	2014.4–2019.1	randomized parallel-group trial	former smokers with moderate-to-severe COPD	85	HEPA	living room, bedroom	6 month intervention	PM_2.5_, PM_10_, PM_2.5–10_, UFP	24 h ambulatory HRV	improved RMSSD
Ahuja et al.^100^	Rohtak, India	2023.11–2024.1	randomized crossover trial	females of an old age home	29	HEPA	room in aged-care center	2 weeks per phase, no washout	PM_2.5_, PM_10_	BP, PWV, CRP, 8-oxo-DG	significant improvement
Arya et al.^82^	Rohtak, India	2023.11–2024.1	randomized crossover trial	male children of an orphanage	32	HEPA	dormitory	2 weeks per phase, no washout	PM_2.5_, PM_10_	BP, PR, CRP, PWV, 8-oxo-DG	significant improvement
Brugge et al.^101^	Massachusetts, US	2020–2024	randomized crossover trial	adults living near major highways	154	HEPA	living room, bedroom	1 month per phase, 1 month washout	PM_2.5_, UFP	BP	significant improvement
Young et al.^103^	Seattle, US	2014–2016	randomized double-blind crossover trial	normotensive adults	13	in-vehicle HEPA filtration	in-vehicle	2 h drives per phase, ≥ 3 week washout	PM_2.5_, BC, PNC	BP, central retinal arteriolar equivalents	improved SBP
Hudda et al.^102^	Boston and Somerville, US		randomized double-blind crossover trial	adults	77	HEPA	room near highway	2 h per phase, 1 week washout	PNC, BC	BP, HR	improved SBP

a Abbreviations: HEPA, high-efficiency particulate air; PM_2.5_, particulate matter with an aerodynamic diameter ≤ 2.5 μm; PM_10_, particulate matter with an aerodynamic diameter ≤ 10 μm; PM_1_, particulate matter with an aerodynamic diameter ≤ 1 μm; PM_2.5–10_, particulate matter with an aerodynamic diameter between 2.5 and 10 μm; UFP, ultrafine particles; BC, black carbon; PNC, particle number concentration; VOC, volatile organic compounds; CO_2_, carbon dioxide; BP, blood pressure; SBP, systolic blood pressure; HR, heart rate; PR, pulse rate; HRV, heart rate variability; RMSSD, root-mean-square of successive difference; ABPM, ambulatory blood pressure monitoring; PWV, pulse wave velocity; MVF, microvascular function; FMD, flow-mediated dilation; CAD, coronary artery disease; COPD, chronic obstructive pulmonary disease; CRP, C-reactive protein; IL-6, interleukin-6; TNF-α, tumor necrosis factor-α; TNF-RII, tumor necrosis factor receptor II; MDA, malondialdehyde; 8-OHdG, 8-hydroxy-2′-deoxyguanosine; 8-oxo-DG, 8-oxo-7,8-dihydro-2′-deoxyguanosine; 8-iso-PGF_2α_, 8-iso-prostaglandin F_2α_.

**Table 4:** Characteristics of Included Studies on Neurocognition and Children Development, Cost-Effectiveness, and Others^a^

**author(s)**	**study region**	**study period**	**study design**	**study population**	**sample size**	**purifier type**	**setting**	**intervention duration**	**pollutants**	**outcome(s)**	**result(s)**
Lamport et al.^117^	Reading, UK	2021.5–2021.9	randomized double-blind crossover trial	healthy adults	30	HEPA	residence	2 weeks per phase, 2 week washout	PM_2.5_, PM_10_, VOC, NO_2_	daily sleep outcomes and mood, ISI, PSQI, symptoms of anxiety and depression	significant improvement
Xu et al.^114^	Tianjin, China	2019.11.30–2019.12.7	randomized crossover trial	healthy adults	162	HEPA	classroom	intervention throughout each exam period, 1 week washout	PM_10_, PM_2.5_, PM_1_, BC	English exam scores	significant improvement
Zhou et al.^115^	Beijing, China	2022	randomized double-blind crossover trial	healthy adults	55	HEPA	office	1 day per phase (5–7h), no washout	PM_2.5_	cognitive test outcomes	significant improvement
Wargocki et al.^112^	Copenhagen, Denmark Lund, Sweden	winter and early spring in 2005	randomized crossover trial	healthy children	190	electrostatic air purifier	classroom	1 week per phase, no washout	PM_2.5_, UFP	homework performance	no significant change
Wang et al.^55^	Beijing, China	2017.12–2018.4	randomized double-blind crossover trial	healthy adults	24	HEPA	dormitory	pollution wave, >2 week washout	PM_2.5_	plasma metabolome	significant improvement
Chen et al.^61^	Shanghai, China	2015.11–2015.12	randomized double-blind crossover trial	healthy adults	45	HEPA	dormitory	9 days per phase, 12 day washout	PM_2.5_	urinary metabolome	significant improvement
Brugge et al.^96^	Chelsea and Boston, US	2013	randomized double-blind crossover trial	low-income Puerto Rican adults	23	HEPA	living room or bedroom	3 weeks per phase, no washout	UFP	TNF-RII, IL-6, hsCRP	no significant change
Ke et al.^116^	Beijing, China		randomized double-blind crossover trial	healthy adults	93	HEPA	fitness center	2 h intervention	PM_2.5_	cognitive function, transcriptomics, metabolomics IgG, IgA, IgM, TP, GLB, total Tau, and BDNF levels, proteomics	significant improvement
Gignac et al.^111^	Barcelona, Spain	2018.11–2019.6	randomized crossover trial	adolescents	2123	cartridge filter purifier	classroom	1.5 h intervention	PM_2.5_, BC	response speed consistency; impulsivity, selective attention, alerting, orienting, and conflict scores	no significant change
Ulziikhuu et al.^105^	Ulaanbaatar, Mongolia	2014.1.9–2020.1.8	randomized parallel-group trial	children	242	HEPA	main living area, bedroom	deployed during pregnancy at median 10 weeks gestation	PM_2.5_	cognitive performance, IQ	significant improvement susceptible population: mothers who did not use vitamin supplements, experienced higher levels of stress, or had lower educational attainment.
Enkhbat et al.^109^	Ulaanbaatar, Mongolia	2014.1.9–2020.1.8	randomized parallel-group trial	children	387	HEPA	main living area, bedroom	deployed during pregnancy at median 11 weeks gestation	PM_2.5_	SRS score	no significant change
Enkhbat et al.^110^	Ulaanbaatar, Mongolia	2014.1.9–2017.12	randomized parallel-group trial	children	407	HEPA	main living area, bedroom	deployed during pregnancy at median 11 weeks gestation	PM_2.5_	behavior problem scores	no significant change
Tamana et al.^108^	Ulaanbaatar, Mongolia	2014.1.9–2017.12	randomized parallel-group trial	children	480	HEPA	main living area, bedroom	deployed during pregnancy at median 11 weeks gestation	PM_2.5_	BMI z-score	significant improvement
Barn et al.^107^	Ulaanbaatar, Mongolia	2014.1.9–2015.5.1	randomized parallel-group trial	children	463	HEPA	main living area, bedroom	deployed during pregnancy at median 11 weeks gestation	PM_2.5_	birth weight	significant improvement
Ulziikhuu et al.^106^	Ulaanbaatar, Mongolia	2014.1.9–2020.1.8	randomized parallel-group trial	children	383	HEPA	main living area, bedroom	deployed during pregnancy at median 11 weeks gestation	PM_2.5_	full scale IQ	significant improvement
Rosén et al.^113^	Uddevalla, Sweden		randomized parallel-group trial	preschool children	93	electrostatic air purifier	daycare center	1 year intervention	very fine particles, fine particles	sick-day absenteeism rate	significant improvement
Kwag et al.^81^	Seoul and Ulsan, South Korea	2017–2020	randomized double-blind crossover trial	housewives	40	HEPA	residence	12 week intervention	PM_2.5_, PM_10_	hemoglobin, MCV, MCH, MCHC	significant improvement
Zhang et al.^119^	China	2019	modeling study	all-age population			residence	long-term intervention	PM_2.5_	annual operating cost of an air purifier, DALYs	significant cost-effectiveness
Fisk et al.^120^	Elizabeth, Los Angeles, Houston; US	2010–2013	modeling study	all-age population		HVAC system, HEPA	residence	long-term intervention	PM_2.5_	estimated potential mortality reductions from filtration improvements	significant cost-effectiveness
Fisk et al.^124^	six counties in Southern California, US	2003	modeling study	all-age population		HVAC system, HEPA	residence	a 10 day period of wildfire smoke exposure	wildfire-generated PM_2.5_	estimated reductions in hospitalizations (overall respiratory, asthma, bronchitis, COPD, pneumonia) and premature deaths	significant cost-effectiveness
Martenies et al.^121^	Detroit and adjacent cities, US	2011–2015	modeling study	children	136,000	enhanced HVAC filters (MERV 8–14), HEPA	bedroom, living room, classroom	long-term intervention	PM_2.5_	incidence of asthma-related outcomes (hospitalizations, ED visits and symptom days), DALYs, monetized impacts	significant cost-effectiveness in schools
Cooper et al.^122^	UK	2019	modeling study	all-age population		HEPA	residence	long-term intervention	PM_2.5_	mortality and life expectancy	life expectancy increase
Liu et al.^118^	China	2016	modeling study	all-age population	the population of mainland China in 2016	HEPA	residence	1 year intervention	PM_2.5_	avoidable mortality under each intervention scenario (S1–S4)	significant cost-effectiveness
Socolovsky et al.^123^	Boston, US	school year	modeling study	students with asthma in urban schools	154	HEPA	classroom	166 day evaluation period	particulate air, Indoor allergens	QALYs, healthcare utilization, costs	significant cost-effectiveness

a Abbreviations: HEPA, high-efficiency particulate air; HVAC system, heating, ventilation, and air conditioning system; PM_2.5_, particulate matter with an aerodynamic diameter ≤ 2.5 μm; PM_10_, particulate matter with an aerodynamic diameter ≤ 10 μm; PM_1_, particulate matter with an aerodynamic diameter ≤ 1 μm; UFP, ultrafine particles; BC, black carbon; VOC, volatile organic compounds; NO_2_, nitrogen dioxide; IQ, intelligence quotient; BMI, body mass index; MCV, mean corpuscular volume; MCH, mean corpuscular hemoglobin; MCHC, mean corpuscular hemoglobin concentration; ED, emergency department; COPD, chronic obstructive pulmonary disease; CRP, C-reactive protein; IL-6, interleukin-6; TNF-RII, tumor necrosis factor receptor II; IgG, immunoglobulin G; IgA, immunoglobulin A; IgM, immunoglobulin M; TP, total protein; GLB, globulin; BDNF, brain-derived neurotrophic factor; DALYs, disability-adjusted life years; QALYs, quality-adjusted life years; PSQI, Pittsburgh sleep quality index; ISI, insomnia severity index; SRS, social responsiveness scale.

### 3.2 Respiratory Health Benefits from Indoor Air Pollution Reduction via an Air Purifier

Research investigating the respiratory effects of air purifier use has been extensively conducted across diverse populations, involving healthy individuals and those with respiratory diseases, to ascertain whether air purifier can serve as an adjunctive measure for symptom amelioration and disease management (detailed study characteristics shown in Tables 1 and 2).

#### 3.2.1 Effect of Air Purifiers on Respiratory Health among Populations with Respiratory Diseases

Compelling evidence from numerous RCTs confirms that air purification is an effective adjunctive therapy for individuals with conditions, such as asthma, COPD, and allergic rhinitis.^15–29^ The majority of these studies, spanning various global regions and intervention durations, have consistently demonstrated that reducing indoor pollutants and allergens leads to measurable clinical improvements in both children and adults.^15–18,22,25,28,30–42^ The evidence of the benefits of air purification is particularly robust in the context of childhood asthma. Numerous RCTs, primarily conducted in the United States, have documented significant clinical improvement. A landmark year-long trial demonstrated that interventions in children with atopic asthma led to fewer symptomatic days and substantial reductions in home allergen levels.^40^ Analogous positive findings on asthma control have been consistently reported in a large body of other US-based research,^28,33,35,36,38,39,41,42^ with comparable benefits also observed in studies from The Netherlands,^25^ South Korea,^34^ Cyprus, and Greece.^37^ Similarly, interventions in adults with respiratory diseases have yielded similar positive results. For example, a randomized, placebo-controlled trial in the Netherlands involving allergic asthma patients demonstrated that air purifier use improved airway hyperresponsiveness and increased forced expiratory volume.^17^ These general benefits for adults with respiratory conditions have been further corroborated by RCTs conducted in China,^15^ France,^16^ the US,^30^ and the UK.^31^ Some studies also suggested that these benefits extend across the lifespan.^22,32^

However, these health benefits were not uniform across all measured outcomes, suggesting a complex interplay of pathophysiological pathways.^19–21,23,26,43–48^ For instance, a 6 month intervention study in China improved clinical asthma control test scores in children but had no significant effect on FeNO levels.^46^ Three RCTs conducted among former smokers with diagnosed COPD in a US region also indicated that although an air purifier reduced PM_2.5_ concentrations and yielded clinically significant respiratory health improvements,^20,21^ it did not significantly alter biomarkers of systemic inflammation, platelet activation, endothelial dysfunction, or oxidative stress.^19^ Conversely, an RCT in China found that a two week intervention led to significant improvements in FeNO and peak expiratory flow yet failed to produce significant changes in other lung function parameters such as FEV_1_, FVC, or the FEV_1_/FVC ratio.^26^ These findings may suggest that the pathophysiological mechanisms underlying these improvements involve multiple independent pathways.

Furthermore, a minority of RCTs reported no significant respiratory health benefits.^24,27,29,49–52^ These null findings may be attributable to methodological limitations in earlier studies, such as suboptimal purification technology;^27,49,51,52^ meanwhile, other trials may have been impacted by uncontrolled confounding factors, with one study specifically identifying the COVID-19 pandemic as a potential contributor.^50^

#### 3.2.2 Effect of Air Purifiers on Respiratory Health among the Healthy Population

Several studies have investigated the effects of air purifier use on respiratory function and biomarkers in healthy populations, further employing omics to investigate the biological mechanisms linking pollutant reduction to health improvements.^53–67^ The most consistent evidence for respiratory benefits originates from studies conducted in China, across both adult and child populations. A large body of trials focusing on healthy young adults have demonstrated that even short-term air purification can significantly improve lung function and reduce biomarkers of airway inflammation and oxidative stress.^53–57,60,61,64^ For example, a randomized, double-blind crossover trial in Shanghai found that short-term air purification was significantly associated with reductions in several circulating inflammatory biomarkers, including interleukin-1β (IL-1β; −68.1%), myeloperoxidase (−32.8%) and soluble CD40 ligand (sCD40L; −64.9%), as well as FeNO (−17.0%).^57^ Other studies using metabolomics have further suggested that by mitigating PM_2.5_ exposure, air purification may reverse disturbances in energy metabolism and inflammatory responses impacting respiratory health.^55,61^ This positive trend extends to healthy children, with a few RCTs showing significant improvements in lung function and biomarkers of oxidative stress and inflammation after both short- and long-term interventions.^65,66^ However, this largely positive picture is not without exception; one study using a negative ion purifier in adults found no net health benefit, a finding attributed to potential offsetting effects from the technology’s byproducts.^58^

In contrast, findings from other geographical regions are inconsistent. A trial in Canada involving healthy adults reported significant increases in FEV_1_ and PEFR,^59^ while two RCTs in Japan observed no significant respiratory benefits in adults,^62,63^ and a US-based trial that recruited young children from homes with wood-burning stoves found no significant reduction in the rates of lower respiratory tract infections.^67^ This heterogeneity highlights that the efficacy of air purification in healthy individuals may be strongly influenced by regional differences in environmental factors, population characteristics, and specific exposure contexts. Furthermore, emerging technologies such as NTP purifiers have shown promise in reducing the incidence and symptoms of upper respiratory tract infections in office settings, suggesting a potential role in inactivating airborne pathogens.^68^

### 3.3 Cardiovascular Health Benefits from Indoor Air Pollution Reduction via an Air Purifier

Evidence on the cardiovascular benefits of air purifiers is substantial but mixed. While a clear majority of trials, particularly in healthy young adults, report positive outcomes, a minority have documented inconsistent or null findings. Furthermore, for vulnerable groups, such as children and older adults, the evidence remains scarce and often contradictory (detailed study characteristics are shown in Tables 2 and 3).

#### 3.3.1 Effect of Air Purifiers on Cardiovascular Health among Healthy Populations

Among healthy young populations, a strong consensus of positive outcomes has emerged, particularly from studies conducted in China. These trials consistently demonstrated that air purifier use can significantly lower blood pressure (BP)^53,56,57,69,70^ and increase heart rate variability (HRV).^71,72^ Mechanistic investigations have further illuminated the biological pathways that may underpin these clinical improvements. These mechanistic studies have assessed the impact of air purification by measuring changes in a range of well-established targeted biomarkers before and after the intervention. These biomarkers are typically associated with systemic inflammation, oxidative stress, endothelial function, and coagulation, such as C-reactive protein (CRP), 8-hydroxy-2′-deoxyguanosine (8-OHdG), interleukin-6 (IL-6), and tumor necrosis factor alpha (TNF-α). The evidence from these trials suggests that air purification may improve cardiovascular health by mitigating proinflammatory and oxidative responses, reducing endothelial damage, and modulating hormonal axes.^53,54,56,57,60,73–78^ Furthermore, some omics studies indicate that air purification can reverse short-term, PM_2.5_-induced disruptions in broader metabolic pathways and epigenetic modifications related to cardiovascular health.^55,61,78–80^ For example, four randomized crossover double-blind trials conducted in the same Chinese population demonstrated that short-term PM_2.5_ exposure adversely affected cardiovascular metabolic health by disrupting metabolic pathways, activating stress response systems, regulating miRNA-cytokine networks, and inducing abnormal DNA methylation. These findings provide multilevel evidence from metabolic phenotypes to epigenetic modifications, illuminating the biological mechanisms through which PM_2.5_ contributes to cardiovascular diseases.^61,76,77,79^ Further extending this evidence, a 3 month intervention in 40 Korean housewives demonstrated that air purification could also mitigate PM_2.5_-associated reductions in hemoglobin and mean corpuscular hemoglobin concentration (MCHC), suggesting a potential role in reducing the risk of anemia.^81^

#### 3.3.2 Effect of Air Purifiers on Cardiovascular Health among Vulnerable Populations

For children, evidence is emerging but inconsistent. An RCT in an Indian orphanage demonstrated that HEPA filtration during sleep time significantly reduced BP, pulse wave velocity (PWV), and biomarkers of inflammation and DNA damage.^82^ Similarly, a study in a high-pollution region of China (Jiaozuo) found that after more than two months of classroom and bedroom air purification intervention, children’s pulse pressure (PP) was notably reduced (−3.44 mmHg, 95% confidence interval: −8.12, −0.11).^83^ Conversely, another RCT in Beijing school classrooms did not observe improvements in HRV; instead, a negative effect was found.^84^ Subsequent urinary metabolomics analysis suggested that while PM reduction was beneficial, coemitted negative ions from the ionization purifier counteracted these positive effects through different metabolic pathways.^85^ The evidence from studies in children remains insufficient to further confirm the potential cardiovascular benefits of air purifier use, necessitating more research conducted in other regions for further validation.

For older adults, the findings are similarly heterogeneous. While most RCTs from China consistently reported improved cardiovascular health outcomes across various subgroups,^86–92^ a study using PCO and filtration purifiers showed that despite reductions in indoor concentrations of multiple pollutants, there was no significant improvement in BP levels.^93^ Besides, studies from the US, Denmark, South Korea and India have presented a more complex picture. For example, some US-based trials found benefits in certain parameters like SBP^94^ or RMSSD^95^ but not in others like aortic hemodynamics^94^ or cardiovascular-related inflammatory markers.^96^ Similarly, studies in Denmark and South Korea reported either conflicting conclusions^97,98^ or benefits limited to specific markers like baroreflex sensitivity rather than BP.^99^ However, a crossover study conducted in India found that a 2 week HEPA intervention significantly lowered SBP, PWV, CRP, and 8-oxo-2′-deoxyguanosine (8-oxo-DG).^100^ These discrepancies may be related to differences in intervention duration and baseline pollution levels across study regions, but other unobserved factors were also likely to influence the results.

Furthermore, a critical and growing body of evidence addresses populations exposed to traffic-related air pollution (TRAP). A pragmatic randomized crossover trial among adults living near major highways in the US found that air filtration resulted in a clinically significant SBP reduction in individuals with elevated baseline SBP (≥120 mmHg), even in environments with relatively low PM_2.5_ concentrations.^101^ Complementing this, another RCT showed that reducing indoor TRAP concentrations with HEPA filters prevented acute, dose-dependent increases in SBP over a 2 h period.^102^ Besides, Young et al. found that in-vehicle HEPA filtration mitigated the adverse effects of TRAP on BP at 1 and 24 h postexposure.^103^

### 3.4 Benefits of Neurocognitive and Children Development from Indoor Air Pollution Reduction via an Air Purifier

As emerging studies have gradually revealed associations between air pollution and neurodevelopment, cognitive performance, and academic achievement,^104^ some research has begun to explore whether improving air quality can benefit neurodevelopmental processes or enhance cognitive-behavioral abilities. Current studies primarily focus on children and adolescents, who are in critical developmental stages and thus are more vulnerable to environmental risks (detailed study characteristics are shown in Table 4).

#### 3.4.1 Effect of Air Purifiers on Neurocognitive Health and Development among Children and Adolescents

Allen and colleagues conducted the “Ulaanbaatar Gestation and Air Pollution Research” (UGAAR) study in Mongolia, investigating the multifaceted effects of prenatal air purifier use on fetal development and subsequent child outcomes. Utilizing a parallel controlled design, the study tracked pregnant women using true or sham air purifiers and compared their offspring across multiple domains, including cognitive development and performance, BMI, autistic behaviors, behavioral problem scores, and fetal growth, to assess potential prenatal health benefits. The study revealed that reducing prenatal PM_2.5_ exposure via air purification promoted child brain development,^105^ and improved cognitive ability at age four.^106^ Such interventions also increased birth weight in full-term infants,^107^ and potentially improved childhood cardiometabolic health.^108^ However, the overall impact on childhood autism-related behavior scores was limited,^109^ with no significant effect on behavioral problem scores.^110^ Additionally, air purification intervention trials in schools across Spain, Denmark, and Sweden showed that despite significant reductions in indoor pollutants, short-term purification did not affect attention function,^111^ academic performance, and environmental perception^112^ in children and adolescents. It is postulated that the intervention duration may have been too brief or that the intervention did not occur during the critical early prenatal period, precluding observation of potential benefits. Conversely, a two year purification intervention found that reducing airborne particulate levels significantly decreased the rate of illness-related absenteeism by 55% among children in two large Swedish daycare centers.^113^

#### 3.4.2 Effect of Air Purifiers on Neurocognitive Health among Adults

Limited research has also focused on cognitive performance and sleep in healthy adults using air purifiers. Four RCTs from China consistently demonstrated that short-term air purification enhanced exam scores,^114^ significantly improved cognitive performance,^71,115^ with particular benefits observed in the memory domain.^115^ Mediation analyses indicated that the neurotoxicity of particulate matter may impair cognitive processing speed via oxidative stress pathways,^114^ enhance information processing speed and reduce error rates in executive function,^115^ and potentially impact cognitive processing efficiency through the “cardio–pulmonary–brain axis” mechanisms mediated by the autonomic nervous system.^71^ Further multiomics evidence confirms that air purification can counteract PM_2.5_-induced cognitive impairment linked to hypoxia, mitochondrial dysfunction, and impaired immune responses.^116^

Beyond cognitive improvements, an RCT in the UK showed that short-term (2 week) home air purification improved nocturnal sleep duration and total time in bed, although no significant improvement in sleep efficiency was observed.^117^ Notably, this effect was only apparent when the experimental group received the “sham-purifier” sequence first, suggesting that future research in this area should consider incorporating appropriate adaptation phases.

### 3.5 Cost-Benefit Analysis of Air Purification and Associated Health Benefits

Several modeling studies have systematically examined the aggregate health benefits and economic feasibility of air purifier deployment, primarily in China, the US, and the UK (Table 4). These analyses consistently conclude that air purification is an economically viable public health intervention with health-related economic benefits often substantially outweighing implementation costs, particularly when high-efficiency filtration is used.

Key findings from studies in China project that widespread air purifier implementation could avert tens of thousands of premature deaths and millions of DALYs annually.^118,119^ Similarly, analyses from the US and UK indicate that enhancing air filtration in residential, school, and commercial buildings could significantly reduce PM-related mortality and asthma burdens while increasing national life expectancy by a cumulative 23 million life-years gained over a lifetime.^120–123^ A consistent theme across these studies is that the net monetary benefit is greatest under moderate pollution reduction targets and that interventions in schools may offer superior cost-effectiveness compared to residential settings.^121,123^ However, the cost-benefit calculus is not universally positive and depends heavily on the context. For instance, an analysis of filtration interventions during wildfire events in Southern California found that while they effectively reduced hospitalizations and deaths, the full implementation costs could exceed the economic savings from averted hospitalizations. In such acute scenarios, the intervention only became cost-effective when accounting for the economic value of averted mortality, particularly when targeted at vulnerable populations, such as the elderly.^124^ This highlights the critical need for further research to identify optimal cost-benefit ratios and target populations to maximize the public-health impact of air purification strategies.

## 4 Limitation, Challenge, and Prospect

Although air purifiers demonstrate considerable promise, their translation into a globally effective public health tool is impeded by significant gaps in scientific evidence and formidable real-world barriers to their implementation. Overcoming these interconnected obstacles is essential to realizing the full potential of this technology and achieving tangible health gains.

A primary limitation is the unbalanced composition of the current evidence base. The vast majority of existing research originates from urban settings in middle- and high-income countries. However, recent research has begun to address this gap. Specifically, Ahuja et al.^100^ and Arya et al.^82^ provide crucial evidence from northern India, demonstrating significant cardiovascular benefits for both elderly and pediatric populations in an environment with hazardous PM_2.5_ levels. These studies validate the efficacy of air purifiers in some of the world’s most polluted regions. Despite this progress, a critical knowledge gap remains concerning their feasibility and long-term effectiveness in diverse low-income and rural populations globally. Future research must continue to strategically prioritize these vulnerable groups to generate context-specific evidence where the potential public health impact is greatest. This need is further underscored by substantial geographic and socioeconomic disparities that limit the global representativeness of the current evidence base. The majority of included studies are concentrated in East Asia, North America, and Europe, with a notable lack of robust interventional data from low- and middle-income countries, particularly in Africa. Given the high burden of household air pollution in these regions, future epidemiological research should prioritize these underrepresented settings to inform equitable and context-specific public health interventions.

The field is further challenged by a lack of methodological uniformity. Profound heterogeneity across study designs (including wide variations in intervention durations, purifier technologies, and assessed health outcomes) complicates the synthesis of findings and hinders direct comparisons. This situation highlights an urgent need for the development of standardized research protocols. An essential consideration in developing these protocols is the recognition of seasonal variability, which can inherently confound the efficacy of indoor air filtration. Ventilation behaviors usually vary across seasons, such as the practice of sealing windows during winter heating periods and the reliance on air conditioning systems during summer. This heterogeneity significantly alters the air exchange rate. Future long-term interventions should consider monitoring window-opening behaviors or stratifying health outcomes by season to accurately assess purification efficacy. Moreover, the research agenda must evolve to rigorously evaluate emerging technologies beyond HEPA filtration. For instance, the recent trial on NTP suggests a promising avenue for reducing respiratory infection incidence in office settings. However, such novel technologies require thorough investigation into their long-term effectiveness and safety profiles to ensure they do not introduce harmful byproducts, a known concern with older ionization technologies.^125^ The establishment of rigorous, universal standards for safety and performance, including clear metrics like the clean air delivery rate (CADR), remains essential for all purifier types. Furthermore, the research agenda should also expand to quantitatively evaluate the benefits of reducing other hazardous indoor pollutants, notably formaldehyde. To inform effective public health guidance, future research and implementation strategies must emphasize the critical role of device sizing and spatial placement. The CADR of a purifier must be appropriately matched to the volume of the room. If a device is undersized for the space, the air exchange rate will be insufficient, which reduces the overall effectiveness of the intervention regardless of manufacturer-specified CADR. Optimal device placement is also critical for maximizing health benefits. Devices should be used in areas of prolonged occupancy (e.g., bedrooms) and positioned centrally without obstructions to ensure effective air circulation. Future trials and guidelines should report room volume relative to CADR, standardize placement, and offer evidence-based recommendations on device sizing. Furthermore, a critical yet frequently overlooked physical limitation of air purifier interventions is acoustic disturbance. Chronic exposure to such nocturnal noise is a well-documented environmental stressor that can induce sleep fragmentation and autonomic arousal.^126,127^ Consequently, this acoustic burden may attenuate the cardiovascular and neurocognitive benefits derived from improved air quality.^128^ Furthermore, noise-induced annoyance also severely compromises long-term intervention adherence. In real-world settings, users frequently downgrade the fan to “sleep” or “low-speed” modes to mitigate noise. This behavioral adaptation significantly decreases effective CADR, which consequently reduces the overall purification efficiency and may compromise the intended health benefits of the intervention. Future field trials should incorporate objective acoustic monitoring and evaluate the net clinical benefit of these devices under tolerable noise thresholds. Even with a robust evidence base, widespread adoption is obstructed by significant socioeconomic and behavioral hurdles that threaten to widen health inequities. The high initial and maintenance costs of quality devices create a steep accessibility gradient, marginalizing the populations most susceptible to air pollution. A comprehensive strategy is required to dismantle these barriers. Supportive policy initiatives, such as targeted subsidies, are vital for ensuring equitable access. Simultaneously, sustained investment in technological innovation can help lower costs and enhance efficiency. These efforts must be complemented by large-scale public health education programs designed to improve awareness and promote consistent use, ultimately paving the way for broader and more impactful implementation. Finally, the synthesis of current evidence might be subject to publication bias. Therefore, findings should be interpreted with caution.

## 5 Conclusions

Air purifiers, particularly those with HEPA filtration, have been demonstrated to have measurable benefits on respiratory and cardiovascular health and represent a promising, cost-effective strategy to mitigate the health burden of indoor air pollution. However, their potential has not been fully realized due to challenges related to access, adoption, and technological limitations. Future research and supportive policies are needed to address these challenges, thereby enhancing effectiveness and ensuring equitable health benefits, especially for vulnerable populations.

## Data Availability

Data will be made available on request.
